# Supplementary material for: Novice assessors demonstrate good intra-rater agreement and reliability when determining pressure pain thresholds; a cross-sectional study
Source: PeerJ. 2023 Jan 4;11:e14565. doi: 10.7717/peerj.14565 (PMC9825054; doi:10.7717/peerj.14565)
Supplement: Supplemental Information 3 — kPa - kilo Pascal. [file peerj-11-14565-s003.docx]

**Appendix A** – Individual intra-rater between-session agreement using the Somedic algometer

| Tibialis anterior |  |  | Between-session agreement | |
| --- | --- | --- | --- | --- |
| Rater | Subsample size | Mean PPT (kPa) | Standard Error of Measurement | Coefficient of Variation |
| 1 | 11 | 557 | 74 (54 - 117) | 13.2% (9.8% - 21.1%) |
| 2 | 10 | 519 | 83 (61 - 137) | 16.0% (11.7% - 26.3%) |
| 3 | 13 | 584 | 87 (66 - 132) | 14.9% (11.3% - 22.6%) |
| 4 | 12 | 570 | 77 (57 - 119) | 13.4% (10.0% - 20.8%) |
| 5 | 9 | 531 | 47 (34 - 81) | 8.9% (6.4% - 15.2%) |
| 6 | 5 | 584 | 27 (18 - 65) | 4.7% (3.0% - 11.1%) |
| 7 | 7 | 480 | 50 (34 - 95) | 10.3% (7.1% - 19.8%) |
| 8 | 6 | 449 | 55 (37 - 115) | 12.3% (8.3% - 25.7%) |
| 9 | 6 | 818 | 74 (50 - 155) | 9.0% (6.1% - 18.9%) |
| 10 | 6 | 439 | 81 (54 - 169) | 18.4% (12.4% - 38.4%) |
| *Total Group* | *85* | *553* | *71 (64 - 82)* | *12.9% (11.5% - 14.8%)* |
|  |  |  |  |  |
| Rectus femoris |  |  | Between-session agreement | |
| Rater | Subsample size | Mean PPT  (kPa) | Standard Error of Measurement | Coefficient of Variation |
| 1 | 11 | 547 | 37 (27 - 59) | 6.8% (5.0% - 10.8%) |
| 2 | 10 | 561 | 76 (55 - 124) | 13.5% (9.8% - 22.2%) |
| 3 | 13 | 581 | 58 (44 - 87) | 9.9% (7.5% - 15.0%) |
| 4 | 12 | 671 | 71 (53 - 109) | 10.5% (7.9% - 16.3%) |
| 5 | 9 | 598 | 56 (40 - 95) | 9.3% (6.7% - 15.9%) |
| 6 | 5 | 573 | 45 (29 - 106) | 7.8% (5.1% - 18.5%) |
| 7 | 7 | 491 | 52 (36 - 100) | 10.6% (7.3% - 20.4%) |
| 8 | 6 | 545 | 71 (47 - 147) | 13% (8.7% - 27.1%) |
| 9 | 6 | 700 | 73 (43 - 132) | 9.0% (6.1% - 18.9%) |
| 10 | 6 | 413 | 35 (23 - 73) | 8.4% (5.7% - 17.6%) |
| *Total Group* | *85* | *575* | *60 (53 – 68)* | *10.4% (9.2% - 11.9%)* |
|  |  |  |  |  |
| Extensor carpi radialis brevis |  |  | Between-session agreement | |
| Rater | Subsample size | Mean PPT  (kPa) | Standard Error of Measurement | Coefficient of Variation |
| 1 | 11 | 396 | 57 (42 - 91) | 14.4% (10.6% - 22.9%) |
| 2 | 10 | 340 | 51 (37 - 84) | 15.1% (11% - 24.8%) |
| 3 | 14 | 455 | 68 (51 - 100) | 14.8% (11.3% - 22%) |
| 4 | 12 | 421 | 51 (38 - 78) | 12.0% (9.0% - 18.6%) |
| 5 | 9 | 466 | 74 (54 - 127) | 16.0% (11.5% - 27.4%) |
| 6 | 5 | 488 | 42 (27 - 99) | 8.5% (5.5% - 20.2%) |
| 7 | 7 | 278 | 39 (27 - 75) | 14.1% (9.8% - 27.1%) |
| 8 | 6 | 301 | 53 (36 - 111) | 17.6% (11.8% - 36.7%) |
| 9 | 7 | 471 | 80 (56 - 154) | 17.1% (11.8% - 32.7%) |
| 10 | 6 | 224 | 40 (27 - 83) | 17.7% (11.9% - 37.0%) |
| *Total Group* | *87* | *393* | *63 (56 - 72)* | *16.1% (14.3% - 18.4%)* |
|  |  |  |  |  |
| Paraspinal muscles C5-C6 |  |  | Between-session agreement | |
| Rater | Subsample size | Mean PPT  (kPa) | Standard Error of Measurement | Coefficient of Variation |
| 1 | 11 | 319 | 44 (33 - 70) | 13.8% (10.2% - 22%) |
| 2 | 10 | 336 | 26 (19 - 43) | 7.7% (5.6% - 12.7%) |
| 3 | 14 | 336 | 27 (21 - 41) | 8.1% (6.2% - 12.1%) |
| 4 | 12 | 329 | 37 (28 - 57) | 11.2% (8.4% - 17.4%) |
| 5 | 9 | 327 | 33 (24 - 57) | 10.2% (7.3% - 17.5%) |
| 6 | 5 | 356 | 53 (34 - 126) | 14.9% (9.7% - 35.3%) |
| 7 | 7 | 241 | 28 (19 - 53) | 11.4% (7.9% - 21.9%) |
| 8 | 6 | 294 | 41 (27 - 85) | 13.9% (9.3% - 29.0%) |
| 9 | 7 | 391 | 29 (20 - 56) | 7.4% (5.1% - 14.2%) |
| 10 | 6 | 184 | 32 (22 - 68) | 17.6% (11.8% - 36.8%) |
| *Total Group* | *87* | *316* | *36 (32 - 41)* | *11.3% (10.1% - 13.0%)* |

kPa, kilo Pascal.
