# Supplementary material for: Novice assessors demonstrate good intra-rater agreement and reliability when determining pressure pain thresholds; a cross-sectional study
Source: PeerJ. 2023 Jan 4;11:e14565. doi: 10.7717/peerj.14565 (PMC9825054; doi:10.7717/peerj.14565)
Supplement: Supplemental Information 4 — kPa - kilo Pascal. [file peerj-11-14565-s004.docx]

**Appendix B** – Individual intrarater between-session agreement using the Wagner algometer

| Tibialis anterior |  |  | Between-session agreement | |
| --- | --- | --- | --- | --- |
| Rater | Subsample size | Mean PPT  (kPa) | Standard Error of Measurement | Coefficient of Variation |
| 1 | 14 | 477 | 60 (46 - 89) | 12.6% (9.6% - 18.7%) |
| 2 | 12 | 652 | 93 (70 - 144) | 14.3% (10.7% - 22.1%) |
| 3 | 6 | 595 | 21 (14 - 45) | 3.6% (2.4% - 7.5%) |
| 4 | 7 | 559 | 69 (47 - 132) | 12.3% (8.5% - 23.6%) |
| 5 | 10 | 495 | 65 (47 - 107) | 13.1% (9.6% - 21.5%) |
| 6 | 12 | 645 | 115 (86 - 178) | 17.8% (13.3% - 27.6%) |
| 7 | 9 | 693 | 80 (57 - 136) | 11.5% (8.3% - 19.7%) |
| 8 | 9 | 588 | 75 (54 - 129) | 12.8% (9.2% - 21.9%) |
| 9 | 7 | 518 | 34 (23 - 65) | 6.5% (4.5% - 12.5%) |
| *Total Group* | *86* | *579* | *76 (67 - 87)* | *13.1% (11.6% - 15.0%)* |
|  |  |  |  |  |
| Rectus femoris |  |  | Between-session agreement | |
| Rater | Subsample size | Mean PPT  (kPa) | Standard Error of Measurement | Coefficient of Variation |
| 1 | 14 | 532 | 56 (42 - 83) | 10.4% (8% - 15.5%) |
| 2 | 12 | 736 | 72 (54 - 112) | 9.8% (7.3% - 15.2%) |
| 3 | 6 | 777 | 64 (43 - 133) | 8.2% (5.5% - 17.2%) |
| 4 | 7 | 669 | 37 (25 - 71) | 5.5% (3.8% - 10.6%) |
| 5 | 10 | 539 | 52 (38 - 86) | 9.7% (7.1% - 16.0%) |
| 6 | 11 | 643 | 74 (55 - 118) | 11.5% (8.5% - 18.4%) |
| 7 | 9 | 640 | 62 (44 - 106) | 9.7% (6.9% - 16.5%) |
| 8 | 9 | 822 | 44 (32 - 76) | 5.4% (3.9% - 9.3%) |
| 9 | 7 | 632 | 66 (45 - 126) | 10.4% (7.2% - 19.9%) |
| *Total Group* | *85* | *655* | *62 (55 - 71)* | *9.4% (8.4% - 10.8%)* |
|  |  |  |  |  |
| Extensor carpi radialis brevis |  |  | Between-session agreement | |
| Rater | Subsample size | Mean PPT  (kPa) | Standard Error of Measurement | Coefficient of Variation |
| 1 | 14 | 332 | 46 (35 - 69) | 13.9% (10.6% - 20.7%) |
| 2 | 11 | 380 | 32 (23 - 50) | 8.3% (6.1% - 13.2%) |
| 3 | 6 | 445 | 15 (10 - 32) | 3.4% (2.3% - 7.1%)^a^ |
| 4 | 7 | 368 | 31 (21 - 59) | 8.3% (5.8% - 16.0%) |
| 5 | 10 | 312 | 34 (25 - 56) | 10.8% (7.9% - 17.8%) |
| 6 | 12 | 372 | 32 (24 - 49) | 8.5% (6.4% - 13.2%) |
| 7 | 9 | 365 | 51 (36 - 87) | 13.9% (10% - 23.8%) |
| 8 | 9 | 434 | 69 (49 - 118) | 15.9% (11.4% - 27.2%) |
| 9 | 7 | 304 | 81 (56 - 154) | 26.5% (18.3% - 50.8%) |
| *Total Group* | *85* | *364* | *47 (42 - 54)* | *12.9% (11.5% - 14.8%)* |
|  |  |  |  |  |
| Paraspinal muscles C5-C6 |  |  | Between-session agreement | |
| Rater | Subsample size | Mean PPT  (kPa) | Standard Error of Measurement | Coefficient of Variation |
| 1 | 14 | 278 | 36 (28 - 54) | 13.1% (10% - 19.4%) |
| 2 | 12 | 410 | 51 (38 - 79) | 12.4% (9.2% - 19.2%) |
| 3 | 7 | 344 | 64 (44 - 123) | 18.7% (12.9% - 35.9%) |
| 4 | 7 | 287 | 63 (44 - 121) | 22.1% (15.2% - 42.3%) |
| 5 | 10 | 247 | 21 (15 - 35) | 8.5% (6.2% - 14.0%) |
| 6 | 12 | 347 | 40 (30 - 62) | 11.6% (8.7% - 17.9%) |
| 7 | 9 | 329 | 74 (53 - 127) | 22.5% (16.2% - 38.5%) |
| 8 | 9 | 407 | 46 (33 - 79) | 11.4% (8.2% - 19.5%) |
| 9 | 7 | 248 | 24 (17 - 46) | 9.7% (6.7% - 18.6%) |
| *Total Group* | *87* | *325* | *47 (42 - 54)* | *14.6% (13.0% - 16.7%)* |

kPa, kilo Pascal.
