# Supplementary material for: Novice assessors demonstrate good intra-rater agreement and reliability when determining pressure pain thresholds; a cross-sectional study
Source: PeerJ. 2023 Jan 4;11:e14565. doi: 10.7717/peerj.14565 (PMC9825054; doi:10.7717/peerj.14565)
Supplement: Supplemental Information 5 — a Ten raters, six female, four male. kPa - kilo Pascal [file peerj-11-14565-s005.docx]

**Appendix C** – Reproducibility parameters for females and males.

| **Somedic algometer** |  | Between-session  agreement | Between-session reliability |
| --- | --- | --- | --- |
|  | Mean PPT (kPa) | Standard Error of Measurement (kPa) | Intraclass Correlation Coefficient (1,1) |
| Tibialis anterior |  |  |  |
| Female raters^a^ | 522 | 71 (61 – 85) | 0.89 (0.83 – 0.94) |
| Male raters^a^ | 595 | 72 (61 – 90) | 0.93 (0.87 – 0.96) |
| Rectus femoris |  |  |  |
| Female raters | 576 | 66 (57 – 80) | 0.92 (0.85 – 0.95) |
| Male raters | 573 | 51 (43 – 64) | 0.95 (0.90 – 0.97) |
| Extensor carpi radialis brevis |  |  |  |
| Female raters | 380 | 62 (53 – 75) | 0.91 (0.84 – 0.95) |
| Male raters | 410 | 65 (55 – 80) | 0.87 (0.77 – 0.93) |
| Paraspinal muscles C5-C6 |  |  |  |
| Female raters | 310 | 38 (32 – 45) | 0.93 (0.88 – 0.96) |
| Male raters | 324 | 34 (29 – 42) | 0.92 (0.86 – 0.96) |
|  |  |  |  |
| **Wagner algometer** |  |  |  |
| Tibialis anterior |  |  |  |
| Female raters | 594 | 88 (76 – 106) | 0.85 (0.74 – 0.91) |
| Male raters | 559 | 56 (47 – 70) | 0.94 (0.88 – 0.97) |
| Rectus femoris |  |  |  |
| Female raters | 681 | 62 (53 – 74) | 0.94 (0.90 – 0.97) |
| Male raters | 619 | 63 (52 – 78) | 0.95 (0.91 – 0.98) |
| Extensor carpi radialis brevis |  |  |  |
| Female raters | 372 | 42 (36 – 51) | 0.90 (0.83 – 0.94) |
| Male raters | 354 | 53 (45 – 67) | 0.90 (0.81 – 0.95) |
| Paraspinal muscles C5-C6 |  |  |  |
| Female raters | 345 | 45 (39 – 54) | 0.89 (0.81 – 0.93) |
| Male raters | 297 | 51 (43 – 63) | 0.84 (0.72 – 0.92) |

^a^ Ten raters, six female, four male. kPa - kilo Pascal
